# Proteomic Responses of *Roseobacter litoralis* OCh149 to Starvation and Light Regimen

**DOI:** 10.1264/jsme2.ME12029

**Published:** 2012-10-05

**Authors:** Rui Zong, Nianzhi Jiao

**Affiliations:** 1 State Key Laboratory of Marine Environmental Science, Xiamen University, Xiamen, 361005, PR China

**Keywords:** *Roseobacter litoralis*, proteomic analysis, carbohydrate metabolism, transporter, light regimen

## Abstract

*Roseobacter litoralis* OCh149 is a type strain of aerobic anoxygenic phototrophic bacteria in marine *Roseobacter* clade. Its full genome has been sequenced; however, proteomic research, which will give deeper insights into the environmental stimuli on gene expression networks, has yet to be performed. In the present study, a proteomic approach was employed to analyze the status of *R. litoralis* OCh149 in carbon starvation during the stationary phase and its responses to a dark/light regimen (12 h:12 h) in both exponential and stationary phases. LC-MS/MS-based analysis of highly abundant proteins under carbon starvation revealed that proteins involved in transport, the transcription/translation process and carbohydrate metabolism were the major functional categories, while poly-β-hydroxyalkanoate (PHA), previously accumulated in cells, was remobilized after stress. Glucose, as the sole carbon source in the defined medium, was broken down by Entner-Doudoroff and reductive pentose phosphate (PP) pathways. Carbohydrate catabolism-related proteins were down-regulated under light regardless of the growth phase, probably due to inhibition of respiration by light. In contrast, responses of amino acid metabolisms to light regimen varied among different proteins during growth phases depending on cellular requirements for proliferation, growth or survival. Fluorescence induction and relaxation measurements suggested that functional absorption cross-sections of the photosynthetic complexes decreased during the dark period and always recovered to about the previous level during the light period. Although the photosynthetic genes in *R. litoralis* OCh149 are located on the plasmid, these data indicate the regulatory mechanism of photoheterotroph metabolism by both carbon and light availability.

On the ocean surface, 15%–25% of microbial communities consist of the *Roseobacter* clade, occupying diverse marine habitats (6, 7, 55). Some members of this clade are aerobic anoxygenic phototrophic bacteria (AAPB), which possess bacteriochlorophyll *a* (BChl *a*) and utilize sunlight as additional energy for their heterotrophic growth (2, 25). In addition to their ecological significance and biogeographic properties, a wide range of physiological features and capabilities have been revealed, such as symbiosis with algae (54), degradation of dimethylsulfoniopropionate (20), CO oxidation (32), and denitrification (16). Including generalists as well as specialists, the clade plays a significant role in marine biogeochemical cycles (35). Over the past two decades, from the first strain identified to the first genome sequenced, a great deal of knowledge has been gained for a better understanding of their enormous success in marine environments (32, 46). So far, 39 genomes of *Roseobacter* strains have been completely sequenced and annotated (up to 7 March 2012). Furthermore, some functional post-genomic studies have been carried out to examine predictive frameworks and explore metabolic pathways, such as the proteomic and fluxomic responses of *Phaeobacter gallaeciensis* and *Dinoroseobacter shibae* to different growth phases and the availability of glucose (53, 60). In contrast to the well-studied heterotrophic properties, especially the heterotrophic metabolic pathways of the *Roseobacter* clade, little is known about the proteomic response of photoheterotrophic growth in this clade (4).

Among the AAPB members in the marine *Roseobacter* clade, *Roseobacter litoralis* OCh149 is of special interest for photoheterotrophic studies. Unlike its closest phylogenetic species *R. denitrificans* OCh114, *R. litoralis* OCh149 features *pufLM* genes located on a linear plasmid and a much lower cellular content of BChl *a* (40, 46). The photosynthetic genes in *R. litoralis* OCh149 are obtained presumably through horizontal gene transfer (41). This special genomic organization of *R. litoralis* OCh149, distinct from all other BChl *a* containing members of the *Rosobacter* clade, makes it a specific model for photoheterotrophic metabolism. Considering the unusual location of the photosynthetic genes, we carried out proteomic analysis of *R. litoralis* OCh149 in response to carbon starvation in the stationary growth phase and light regimen in different growth phases, and demonstrated not only its photoheterotrophic property, but also the mechanism involved in starvation- and light-regulated proteins.

## Materials and Methods

### Cultivation of experimental bacterium

The *R. litoralis* OCh149 strain was obtained from the Deutsche Sammlung von Mikroorganismen und Zellkulturen (DSMZ, Braunschweig, Germany). It was maintained and precultured aerobically at 25°C in rich organic medium (58), and stirred at a rate of 120 rpm in the dark. To simplify metabolic process analysis, a reported defined marine minimal medium (MMM) (27, 36) was used in the present study. Cells on entering the exponential phase (1/2 OD_max_) were used to inoculate the main cultures, which were sampled for subsequent analysis. The main cultures were grown in 250 mL Erlenmeyer flasks containing 100 mL MMM. The final concentration of the carbon source in 100 mL MMM was provided by 0.2 g glucose.

Two groups of cells were incubated in a growth chamber (Conviron, Winnipeg, Canada) with four incandescent lamps (low light intensity 30 μE m^−2^ s^−1^): (1) light regimen incubation (light/dark cycle of 12 h:12 h), providing the dark period necessary for the synthesis of BChl *a* to retain the availability of the photosynthetic apparatus (PA); and (2) dark incubation (bottles wrapped tightly with aluminum foil), abolishing photosynthetic activity, as the control. The growth curves (Fig. S1C) of *R. litoralis* OCh149 in both experimental and control groups were determined by measuring optical density at 600 nm. Cells were harvested in either the exponential (after incubation for 118 h) or stationary phase (after incubation for 190 h) by centrifugation at 10,000×*g* for 10 min at 4°C. The resulting pellets were stored at −80°C until proteomic analysis was performed.

### 2D-GE

The cell pellets were suspended in 1 mL lysis buffer (7 M urea, 2 M thiourea, 2% CHAPS, 1% DTT, 4% TRITON-X 100, 2% carrier ampholytes, 5% protease inhibitor cocktail) (17). After incubation at room temperature for 30 min with period vortexing, the suspensions were sonicated on ice and centrifuged. The supernatants were directly taken for measurement of protein concentration with the RC DC kit (Bio-rad Laboratories, Hercules, CA, USA). The 2D-GE analysis was performed as follows: In brief, for isoelectric focusing (IEF), approximately 100 μg protein was loaded onto each precast 18 cm immobilized non-linear pH 4–7 strip (Bio-Rad Laboratories) and the strips were rehydrated for 12 h. IEF was carried out in an Ettan IPG-phor3 System (GE Healthcare, Buckinghamshire, UK). SDS-PAGE was carried out on 11.5% polyacrylamide gels in an Ettan DALT apparatus (GE Healthcare). To analyze the reproducibility of 2D gels, we repeated the experiments three times for each sample. Proteins were visualized using silver staining and scanned with an Image Scanner II system (GE Healthcare). Images were analyzed and quantitatively compared using PDQuest 8.0 Software (Bio-Rad Laboratories). After loading the gel images of each growth phase into PDQuest, the same set of triplicate gels was grouped together to determine the average quantities of the protein spots. There were two groups for each growth phases, the dark group and the light regimen group. The “dark” group was chosen as the control group. Spot intensities were normalized according to the mode “total quantity of valid spots” and analyses were performed in quantitative and qualitative modes. The confidence threshold for up- and down-regulation of protein spots was set at two-fold above or below the spot intensity seen in the control.

### MALDI-TOF/TOF-MS/MS analysis

The in-gel digestion of proteins was performed according to a previous protocol with modifications (45). Protein spots of interest were cut from the silver-stained gels and were destained with a solution of 15 mM potassium ferricyanide and 50 mM sodium thiosulfate (1:1) for 20 min at room temperature. The gel pieces were then washed twice with deionized water, shrunk by dehydration in acetonitrile (ACN), and then swollen in digestion buffer containing 10 mM ammonium bicarbonate and 4 ng μL^−1^ trypsin (Promega, Madison, WI, USA) at 4°C. After 30 min incubation, the pieces were digested for more than 12 h at 37°C. Peptides were extracted twice using 5% trifluoroacetic acid (TFA) in 50% ACN. The extracts were dried under the protection of N_2_. MALDI-TOF/TOF analysis was carried out on an AB Sciex TOF/TOF 5800 (Applied Biosystems/Life Technologies, Carlsbad, CA, USA). Peptides were eluted onto the target with 0.7 μL matrix solution (a-cyano-4-hydroxy-cinnamic acid in 0.1% TFA, 50% ACN). Samples were allowed to air dry before loading them into the mass spectrometer.

### LC-MS/MS analysis

Cellular proteins without fractionation from each treatment were obtained using a gel-free approach and digested according to filter-aided sample preparation as outlined by Jacek *et al.* (57) before being loaded onto nano-LC using an Ettan MDLC capillary LC system (GE Healthcare). Peptides were trapped and captured using a 100 μm i.d.×15 mm long pre-column packed with 200 Å, 5 μm MAGIC C18AQ particles. Peptides were separated on a 75 μm i.d.×150 mm long analytical column packed with 100 Å, 5 μm MAGIC C18AQ particles. Peptides were eluted using an acidified (formic acid, 0.1% [v/v]) water–acetonitrile gradient (5–35% acetonitrile for 60 min, 35%–95% acetonitrile for 35 min) at 250 nL min^−1^. Mass spectrometry was performed on a LTQ-Orbitrap mass spectrometer (Thermo Fisher Scientific, Waltham, MA, USA) with NSI. Data-dependent MS/MS spectra were obtained simultaneously. The heated capillary temperature and spray voltage were 200°C and 2.2 kV, respectively. The MS scan range was 300–2,000 m/z with a resolution R = 60,000 at m/z 400. For each cycle, the five most abundant ions from MS analysis were selected for MS/MS analysis using a collision energy setting of 35%. The dynamic exclusion settings were as follows: repeat count 1, repeat duration 30 s, exclusion duration 180 s. The experiments were repeated twice and these results were combined into the final result.

### MS/MS data analysis

For MALDI-MS/MS spectra, all data searches were performed using MASCOT version 2.1 (Matrix Science, London, UK). The following settings were used: NCBInr database (release 20070627; 4182491 sequences; 1439956234 residues), bacteria, trypsin digestion with one missing cleavage, peptide tolerance of ±0.2 Da, fragment mass tolerance of ±0.3 Da, and possible oxidation of methionine.

For LC-MS/MS spectra, each data set was matched using Bioworks 3.2 software (Thermo Fisher Scientific, Waltham, MA, USA) against the *R. litoralis* OCh149 protein file containing 4,746 protein entries compiled from the annotation of the *R. litoralis* OCh149 genome (downloaded from NCBI on 24 March 2010, http://www.ncbi.nlm.nih.gov/). To estimate the false positive rate (FPR), reverse sequences of the *R. litoralis* OCh149 protein file were added to the database. The SEQUEST parameters were set to allow 0.5 Da fragment ion tolerance and 2 missed internal cleavage sites by trypsin. The results of the SEQUEST search were filtered using the Trans-Proteomic Pipeline 4.2.1 (downloaded from http://tools.proteomecenter.org/TPP.ph) with the minimum Peptide-Prophet and ProteinProphet thresholds set at 0.05 and 0.9, respectively. The criterion for protein identification was that at least one of its unique peptides was identified in three or more spectra or that at least two of its unique peptides were identified in one or more spectra. We applied reverse-database searching to determine the FPR. No sequence from the reversed database was detected in the results.

### Morphological and physiological analysis

Cells of *R. litoralis* OCh149 for ultrastructural analysis were collected by centrifugation at 5,000×*g* for 15 min, and fixed in 0.1 M phosphate buffer (2.5% glutaraldehyde) for 12 h at 4°C. Cells were dehydrated with ethanol and treated for embedment in Spurr resin (Sigma-Aldrich, St. Louis, MO, USA). After ultrathin sectioning, the samples were observed under a transmission electron microscope.

The *in vivo* photophysiological parameter of the functional absorption cross section (σ) was measured using the FIRe fluorometer system (Satlantic, Halifax, Canada) at 12 h intervals of sampling, as described previously (50). Fluorescence signals were separated using the infrared filter (880 nm, 50 nm bandwidth) for BChl *a* fluorescence, and the total duration of signal photosynthetic turnover was 400 μs, which gradually closed the reaction centers, and average signals were obtained from 30 iterations of one sample.

We analyzed the C:N ratio of cells retained on a precombusted glass-fiber filter (Whatman GF/F, Whatman/GE Healthcare, Buckinghamshire, UK) with a Vario EL III elemental analyzer (Elementar Analysensysteme, Hanau, Germany). Triplicate samples were run, and the results were converted and calculated as atoms.

## Results and Discussion

### Abundant proteomic profiles of *R. litoralis* OCh149 in carbon starvation

LC-MS/MS proteomic analysis of the cells harvested in the stationary phase showed that light incubation yielded 710 unique peptides corresponding to 206 proteins, while dark incubation yielded 844 unique peptides corresponding to 219 proteins. Taken together, 271 abundant proteins (154 common proteins shared by both incubations) were identified from 1,119 unique peptides that collectively matched 3,315 tandem spectra.

**Fig. 1:**
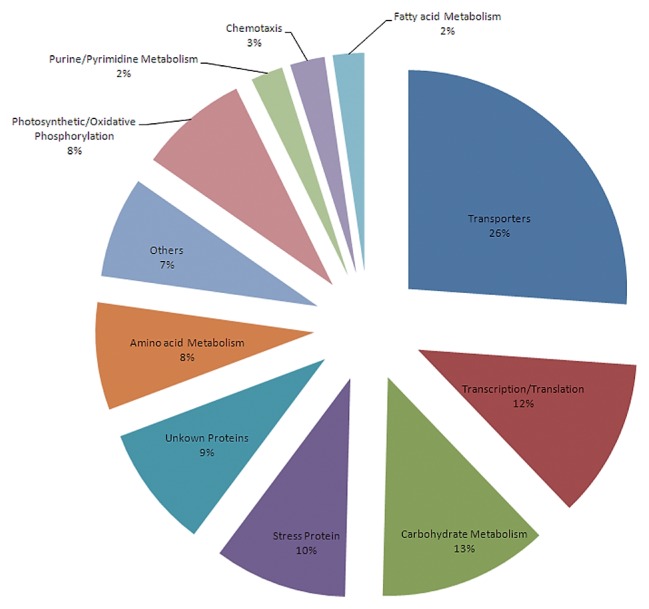
Percentages of the 11 protein groups identified by LC-MS/MS in *R. litoralis* OCh149 in the stationary phase. The functional categories were determined according to the database of the Kyoto Encyclopedia of Genes and Genomes (KEGG). The percentage of each protein group was calculated as follows: the number of tandem spectra of each functional category divided by the number of tandem spectra of total proteins.

All detected proteins were divided into 11 groups by functional category (Fig. 1). Based on the number of tandem spectra, the transporters registered the largest proportion, accounting for 26% of the total. The percentages of housekeeping proteins, including proteins involved in carbohydrate metabolism, transcription/translation process, stress response, amino acid metabolism and photosynthetic/oxidative phosphorylation, ranged from 8% to 13% of the total number of tandem spectra. Other proteins, which were not associated with definite metabolic processes, accounted for 7%. Unknown proteins, expressed by genes encoding hypothetical or conserved hypothetical proteins, amounted to 9% of the detected spectra. The results concerning the proteome in the stationary phase using LC-MS/MS revealed abundant proteins and represented the dominant metabolism of *R. litoralis* OCh149 under carbon-limited conditions.

### Central metabolic pathways of *R. litoralis* OCh149

**Fig. 2:**
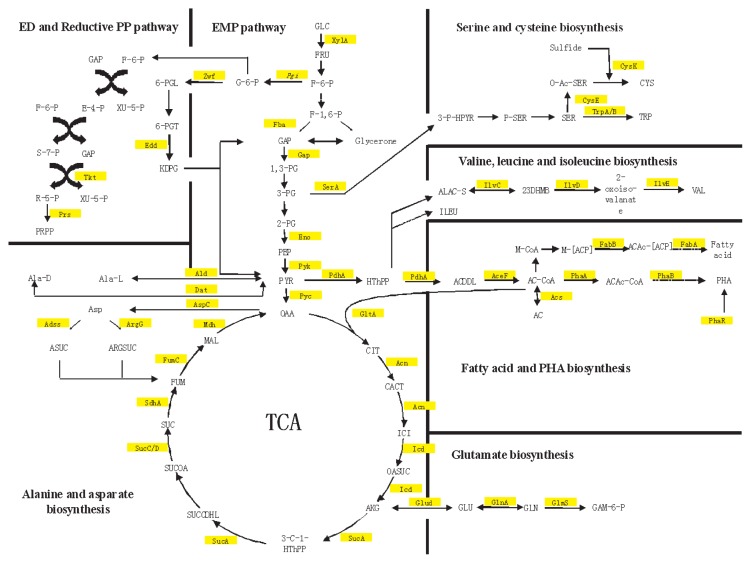
Central metabolic pathways of *R. litoralis* OCh149 in the stationary phase. Enzymes identified using LC-MS/MS are highlighted. Abbreviations (metabolites): GLC, glucose; FRU, fructose; F-6-P, fructose-6P; F-1,6-P, fructose-1,6P; GAP, glyceraldehyde-3P; 1,3-PG, glycerate-1,3P; 3-PG, glycerate-3P; 2-PG, glycerate-2P; PEP, phosphoenol-pyruvate; PYR, pyruvate; OAA, oxaloacetate; CIT, citrate; CACT, cis-aconitate; ICI, isocitrate; OASUC, oxalosuccinate; AKG, 2-oxo-glutarate; 3-C-1-HThPP, 3-carboxy-1-hydroxypropyl-ThPP; SUCCDHL, S-succinyldihydrolipoamide-E; SUCOA, succinyl-CoA; SUC, succinate; FUM, fumarate; MAL, malate; G-6-P, glucose-6P; 6-PGL, 6-phosphogluconolactone; 6-PGT, 6-phosphogluconate; KDPG, 2-dehydro-3-deoxygluconate-6P; E-4-P, erythrose-4P; XU-5-P, xylulose-5P; S-7-P, sedoheptulose-7P; R-5-P, ribose-5P; GLU, glutamate; GLN, glutamine; GAM-6-P, glucosamine-6P; Ala-L, L-alanine; Ala-D, D-alanine; Asp, asparatate; ARGSUC, arginino-succinate; ASUC, adenylo-succinate; 3-P-HPYR, 3P-hydroxy-pyruvate; P-SER, phosphoserine; SER, serine; O-Ac-SER, O-acetyl-serine; CYS, cysteine; TRP, L-tryptophan; ALAC-S, (s)-2-acetolactate; 23DHMB, (R)-2,3-dihydroxy-3-methylbutanoate; ILEU, isoleusine; VAL, valine; HTHPP, 2-hydroxyethyl-ThPP; ACDDL, S-acetyl-dihydrolipoamide-E; AC-CoA, acetyl-CoA; ACAc-CoA, acetoacetyl-CoA; M-CoA, malonyl-CoA; M-[ACP], malonyl-[acp]; ACAc-[ACP], acetoacetyl-[acp]; AC, acetate. Abbreviations (enzymes): XylA, xylose isomerase; Pgi, glucose-6-phosphate isomerase; Zwf, glucose-6-phosphate-1-dehydrogenase; Fba, fructose-bisphosphate aldolase; Gap, glyceraldehyde-3-phosphate dehydrogenase; Eno, enolase; Pyk, pyruvate kinase; Pyc, pyruvate carboxylase; Edd, phosphogluconate dehydratase; Tkt, transketolase; Prs, ribose-phosphate pyrophosphokinase putative; GltA, citrate synthase; Acn, aconitate hydratase; Icd, isocitrate dehydrogenase (NADP-dependent); SucA, alpha-ketoglutarate dehydrogenase; SucC, succinyl-CoA synthetase, beta subunit; SucD, succinyl-CoA synthetase, alpha subunit; SdhA, succinatedehydrogenase flavoprotein subunit; FumC, fumarate hydratase; Mdh, malate dehydrogenase; Ald, alanine dehydrogenase; Dat, D-alanine aminotransferase; AspC, aspartate aminotransferase; Adss, adenylosuccinate synthetase; ArgG, argininosuccinate synthase; CysE, serine acetyltransferase; CysK, O-acetylserine sulfhydrylase; TrpA, tryptophan synthase, alpha subunit; TrpB, tryptophan synthase, beta subunit; IlvC, ketol-acidreductoisomerase; IlvD, dihydroxy-acid dehydratase; IlvE, branched-chain amino acid aminotransferase; Glud, glutamate dehydrogenase; GlnA, glutamine synthetase, type I; GlmS, D-fructose-6-phosphate amidotransferase; PdhA, pyruvate dehydrogenase complex, E1 component, alpha subunit; AceF, dihydrolipoamide acetyltransferase; PhaA, acetyl-CoA acetyltransferase; PhaB, acetoacetyl-CoA reductase; PhaR, polyhydroxyalkanoate synthesis repressor; FabA, 3-hydroxydecanoyl-ACP dehydratase; FabB, 3-oxoacyl-(acyl-carrier-protein) synthase; Acs, acetyl-coenzyme A synthetase. Detailed information about the proteins is provided in supplemental material Table S1.

Based on the abundant proteomic profiles, the central metabolic pathways in *R. litoralis* OCh149 were characterized. This not only allowed improved understanding of the carbon metabolism initiated by glucose, but provided clues to the exploited nutrient metabolism as glucose became gradually unavailable. The deduced central pathways included carbohydrate, amino acid, fatty acid and purine/pyrimidine metabolisms (Fig. 2).

Carbohydrate metabolism is fundamental for bacterial heterotrophic metabolism, providing carbon skeletons, energy and intermediate products. In general, there are three metabolic pathways for the utilization of glucose as a carbon source: the Embden-Meyerhof-Parnas (EMP) pathway, the Entner-Doudoroff (ED) pathway and the pentose phosphate (PP) pathway (oxidative and non-oxidative). In the genome of *R. litoralis* OCh149, all the genes required for the ED pathway are present, while *pfk* (6-phosphofrucokinase) and *pgd* (6-phosphogluconate dehydrogenase) genes, which encode the key enzymes of the EMP and the oxidative PP pathway, respectively, are missing. In this study, Edd, Eda (2-keto-3-deoxy-phosphogluconate aldolase, see the 2-DE results below) and Tkt proteins were identified. The combination of genomic and proteomic data implied that *R. litoralis* OCh149 used the ED pathway for glucose breakdown and the non-oxidative PP pathway for nucleic acid, ATP and coenzymes synthesis.

For comparison of the glucose metabolic pathways in *R. litoralis* OCh149 and other members of the marine *Roseobacter* clade, all 39 available genomes were investigated (Table 1). The non-oxidative PP pathway exists in all 39 known *Roseobacter* genomes and almost all have the *eda* gene, except *Roseovarius nubinhibens* ISM and *Roseovarius* sp. 217. The EMP pathway only exists in *Citreicella* sp. SE45, *Oceanicola granulosus* HTCC2516, *Pelagibaca bermudensis* HTCC2601, *Dinoroseobacter shibae* DFL12, *Maritimibacter alkaliphilus* HTCC2654 and *Sagittula stellata* E-37, with the first two strains possessing all five key genes of the three metabolic pathways, while the EMP pathway has not been reported to be active in AAPB, such as *D. shibae* DFL12, which has been shown to catabolize glucose exclusively via the ED pathway (53). In contrast, some anaerobic anoxygenic phototrophic bacteria (AnAPB) do not possess the related genes in the ED pathway (52). This indicates that the ED pathway is mainly restricted to aerobic prokaryotes (both bacteria and archaea) (8, 15, 51) and members of the *Roseobacter* clade belong to AAPB, which depend on molecular oxygen to synthesize BChl *a*, suggesting that the ED pathway may have a significant role in glucose metabolism in AAPB (53, 60).

**Fig. 3:**
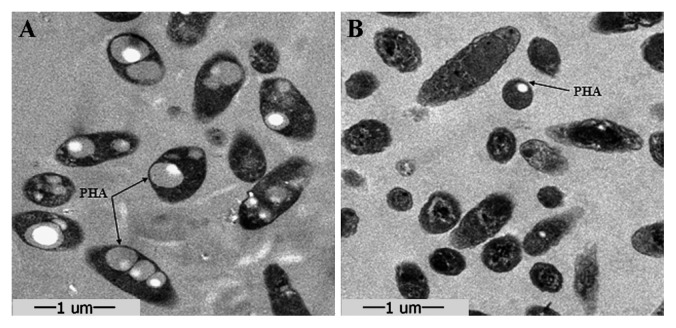
Electron micrographs of ultrathin sections of *R. litoralis* OCh149 and PHA granules. (A) Cells grown in the preliminary medium (rich organic medium). (B) Cells in the late stationary phase in glucose (marine minimal medium).

In order to further examine the central metabolism, the TCA cycle and anaplerotic pathways were reconstructed from the proteomic data. The capability for synthesis of amino acids (alanine, asparate, cysteine, serine and glutamate etc.), fatty acids and poly-β-hydroxyalkanoates (PHAs) was verified (Fig. 2). These substances were converted from glucose and contributed to the dissolved organic carbon pool in the cells; some of the substances could even be transformed to inclusion bodies for carbon storage in the cells (29). For example, PHAs are universal prokaryotic storage material with the proposed designation “carbonosome” (22). In heterotrophic bacteria they are accumulated under unbalanced growth conditions (N or P starvation) when carbon sources are still available, and provide the cells with carbon and energy when the external sources are deficient (60). *R. litoralis* OCh149 proteomic data provided evidence of the enzymes for the synthesis of PHAs. Six genes are involved in PHA biosynthesis and are organized into two distant clusters (*phaAB* and *phaZCPR*) in the *R. litoralis* OCh149 genome. The proteins encoded by three of these appeared in our proteomic data: PhaA, PhaB and PhaR. The existence of PHAs in *R. litoralis* OCh149 was also observed using electron microscopy of ultrathin sections and the identity of PHAs was also confirmed with Nile Blue staining (data not shown). In the cytoplasm of cells from pre-cultivation (rich organic medium), PHA granules were clearly accumulated (Fig. 3A), while in research about phototrophic bacteria, growing photoautotrophically under balanced or unbalanced conditions, glycogen was much more heavily accumulated than PHAs (14). The accumulation of PHAs in *R. litoralis* OCh149 therefore highlighted its heterotrophic characteristics. When *R. litoralis* OCh149 suffered from nutrient limitation in the stationary phase, the previously accumulated PHA granules were found to be smaller or even disappeared from the cells (Fig. 3B). This indicated that PHAs were degraded as carbon and energy sources during starvation; however, PhaZ (polyhydroxyalkanoate depolymerase), responsible for catalyzing the depolymerization of PHAs, was not detected. This could be due to the effects of other molecular mechanisms for PHA degradation. First, PhaR plays a negative role in the regulation of PHA synthesis (12, 28). Excess PhaR can repress the expression of PhaP (Phasin) by binding to upstream DNA regions of *phaP* and *phaR* (39). This could also explain why PhaR was detected, but PhaP was not detected in the present study. Second, PHA-associated proteins may also contribute to PHA degradation. PHAs are complexly organized subcellular structures with many proteins on the granule surface, some of which are essential for PHA metabolism (22). Further work is necessary to reveal the *in vivo* functions of these proteins.

### Transporter proteins in *R. litoralis* OCh149

The large proportion of transporter proteins, as revealed by LC-MS/MS, was a noteworthy finding (Fig. 1). As Gram-negative bacteria, *R. litoralis* OCh149 exchanges substances between the periplasm and cytoplasm by specific substrate-binding transport systems (such as ATP-binding cassette [ABC] transporters, tripartite ATP-independent periplasmic [TRAP] transporters and TonB-dependent [TBD] transporters) and uses porins as molecular sieves in the outer membrane to allow hydrophilic molecules into the periplasmic space (3). In our experiment, three transport systems were detected: ABC transporters, TRAP transporters and porins. Spectra of ABC and TRAP transporters in particular accounted for 20% of the total spectra overall. This number situates *R. litoralis* OCh149 next to a mostly oligotrophic-adapted ubiquitous heterotrophic clade SAR11 (28%–35%) but above the phototrophic representative *Rhodobacter sphaeroides* (11%) (9, 47). This means that the ability of nutrient scavenging in *R. litoralis* OCh149 is more effective than in photoautotrophic bacteria, but less than in typical heterotrophic bacteria. From the viewpoint of physiology, this may be because ATP converted from light energy can substitute for the energy generated from organic substrate oxidation. A transcriptomic study of coastal bacterioplankton showed that *Roseobacter* is the taxon with the most abundant dissolved organic carbon-related transporter sequences (37). *Roseobacter* and SAR11, two clades of ubiquitous and abundant bacterioplankton in the surface ocean (18, 19, 34), possess a high proportion of transporters, which can be interpreted as an advantageous adaptation to diverse marine habitats and an ecologic strategy in low-nutrient environments.

**Fig. 4:**
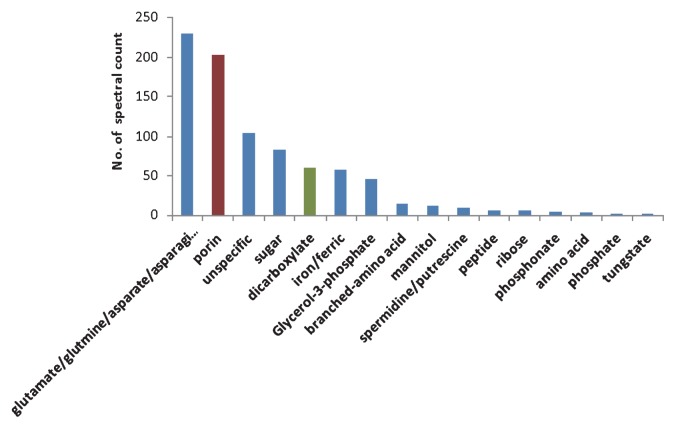
Distribution of spectra from transporters according to the binding substances. Blue column, ABC transporters; green column, TRAP transporters; red column, outer membrane porin proteins.

In addition to abundant transporters, further details on dissolved organic matter (DOM) utilization could be obtained. Among the ABC transporters, extracytoplasmic solute receptors (ESR) were identified, which were involved in the binding of amino acid, branched amino acid, peptide, sugar, mannitol, ribose, phosphate, phosphonate, ferric ion, and tungstate, and among the TARP transporters which were distributed across DctP and TAXI families, only C4-dicarboxylate-binding proteins were detected as ESR (Fig. 4, Table S1). In particular, glutamate/glutamine/aspartate/asparagine-binding protein was the most frequently detected, accounting for 26% of the total transporter spectra. The pathway for the synthesis of glutamate and aspartate from glucose could also be inferred (Fig. 2). This indicated that glutamate or aspartate is an important carbon and nitrogen source for *R. litoralis* OCh149. The dominant uptake of amino acid by *R. litoralis* OCh149 might explain the decrease of the bacterial carbon: nitrogen (C:N) ratio during its growth process (from 5.47±0.33 at the beginning to 3.86±0.02 at the end of culture). Such selective utilization of substrates may also influence the ambient DOM composition, which supports the proposition of the microbial carbon pump in the ocean (23). Transport systems for spermidine/putrescine and dicarboxylate were also detected and were considered to contribute to the uptake of algal osmolytes and the subsequent photo-oxidative products of DOM released by algae (10, 32, 33). *Silicibacter pomeroyi* DSS-3 and *R. litoralis* OCh149 genomes analyses, as genetic proofs, revealed the significant feature of the transport systems for algal osmolytes (13, 32). *Roseobacter* clade members commonly attach to, or live symbiotically with algae in the field (42, 54, 56), such as *R. denitrificans* with *Enteromorpha linza* (46), and *Dinoroseobacter shibae* DFL12 with a dinoflagellate (5). This indicates that *Roseobacter* closely associates with algae by substrate transporters, which are necessary for the transformation and uptake of DOM released from photosynthetically fixed organic carbon by phytoplankton (11).

### Light-induced differential protein expression in different growth phases

**Fig. 5:**
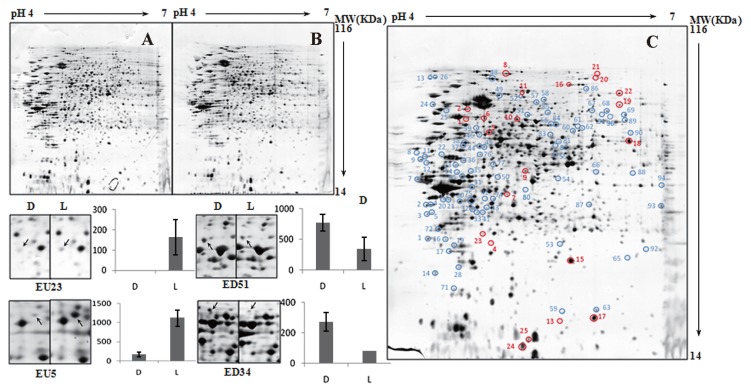
2D-GE image analysis of the *R. litoralis* OCh149 proteome in exponential phase under dark and light regimen treatments. (A, B) A set of two gels from samples treated with dark (A) and light (B) regimens. (C) A representative gel showing the identified differentially expressed proteins. Red, proteins up-regulated in light regimen. Blue, proteins down-regulated in light regimen. (D) Typical examples of spots showing different expression profiles. D, under dark condition. L, under light regimen.

**Fig. 6:**
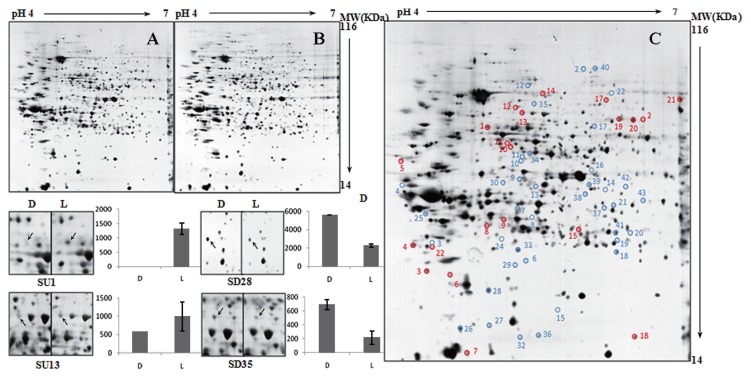
2D-GE image analysis of the *R. litoralis* OCh149 proteome in stationary phase under dark and light regimen treatments. (A, B) A set of two gels from samples treated with dark (A) and light (B) regimen. (C) A representative gel showing the identified differentially expressed proteins. Red, proteins up-regulated in light regimen. Blue, proteins down-regulated in light regimen. (D) Typical examples of spots showing different expression profiles. D, under dark condition. L, under light regimen.

The results of comparative by 2D-GE proteomic analysis of cells harvested in both exponential and stationary phases provided insights into the differences in proteomic responses to light and nutrient stresses (Fig. 5 and 6).

From cells in the exponential phase, 109 proteins were reported to exhibit differential expressions (Table 2), with 24 proteins being up-regulated and 85 proteins down-regulated under the light regimen. In particular, three protein groups were exclusively down-regulated, including transporters (14 proteins), those involved in the photosynthetic/oxidative phosphorylation process (two proteins) and fatty acid metabolism (two proteins), and one protein group involved in chemotaxis (one protein) was exclusively up-regulated. From cells in the stationary phase, 60 proteins were shown to be differentially expressed (Table 3), with 21 proteins being up-regulated and 39 down-regulated under the light regimen. Two protein groups, namely those involved in the stress response (one protein) and fatty acid metabolism (three proteins), were exclusively down-regulated, and one protein group associated with purine/pyrimidine metabolism (one protein) was exclusively up-regulated.

**Fig. 7:**
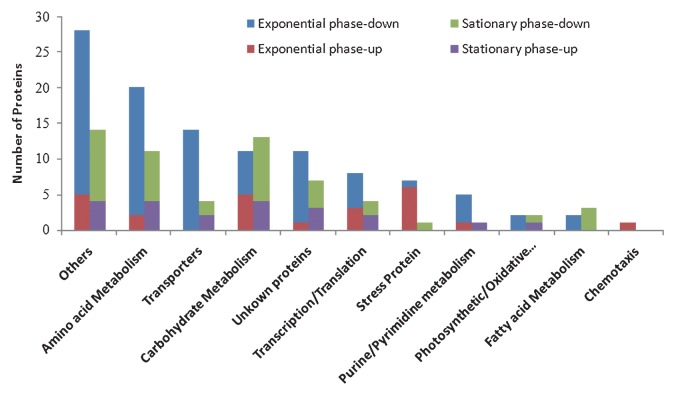
Proteins with differential expression were grouped by functional category. Up-regulated in the light regimen in exponential phase (red), down-regulated in the light regimen in exponential phase (blue), up-regulated in the light regimen in stationary phase (purple), down-regulated in the light regimen in stationary phase (green). Bar heights represent the numbers of proteins.

In five functional categories of metabolism, down-regulated proteins outnumbered up-regulated proteins under the light regimen in both exponential and stationary phases, including amino acid metabolism, carbohydrate metabolism, and fatty acid metabolism, as well as other proteins and unknown proteins (Fig. 7). In some metabolic processes, such as transporters, transcription/translation, photosynthetic/oxidative phosphorylation, down-regulated proteins dominated exclusively in the exponential phase, while stress proteins and chemotaxis proteins were markedly up-regulated. Purine/pyrimidine metabolism-related proteins were mainly down-regulated in the exponential phase but up-regulated in the stationary phase (Fig. 7).

Glycolysis and TCA cycle-related proteins were enhanced in both exponential and stationary phases under dark conditions, and Eda protein, essential for the ED pathway, was also up-regulated in the exponential phase in the dark (Table 2). In addition, the abundance of fructose 1,6-bisphosphate aldolase (Fba), as determined by the above LC-MS/MS results, was higher in the dark (10 spectra) than in the light regimen condition (5 spectra) (Fig. 2, Table S1). These indicated that glucose catabolism was inhibited by light in *R. litoralis* OCh149 due to the common electron carriers shared by photosynthetic and respiratory electron transfer systems (21, 59); however, *Synechocystis* sp. PCC 6803, an oxygenic photoautotroph, exhibits light-activated heterotrophic growth (LAHG) with the induced expression of Fba (49), and LAHG is common to other photoautrophic organisms (61). The differential responses of heterotrophic growth to light between the two phototrophic clades (anoxygenic and oxygenic) were also verified in field research, which demonstrated that light enhanced leucine incorporation by *Cyanobacteria*, while there was no such reaction in AAPB (30). Furthermore, in the present experiment, the synthesis of peptidoglycan (*nagZ*) and lipopolysaccharide (*rfbA*, *xanB*, *kdsD*), the metabolism of gluconeogenesis and other carbohydrates (*tpiA*, *glgC*, *mvaB*, *pgl*, *gtaB*) were also activated in the dark. CO oxidation by members of the widespread *Roseobacter* clade, which is a non-ignorable process in the global carbon cycle, was examined previously through their genome sequences (7, 31, 32, 48). Here, carbon-monoxide dehydrogenase G protein (*coxG*), which can oxidize CO, was detected and repressed by light. Further, by carbonic anhydrase, the production of CO_2_ was attached to PEP, or PYR to form OAA, as a CO_2_ assimilation process.

The response of amino acid metabolism to light varied with the growth phase. In the exponential growth phase, the majority of differentially expressed proteins were down-regulated under light, which participated in the synthesis of serine (*serA/B*), cysteine (*cysE*), tryptophane (*trpC*), leucine (*leuB*), glutamine (*ipuC*), aspartate (*aspB*), threonine (*thrC*), proline (*arcB*), histidine (*hisA*), aromatic (*aroG*) and other branched-chain amino acids (*ilvH*). In the stationary growth phase, the up-regulated proteins were involved in the synthesis of glutamate (*gill*), histidine (*hisA*) and aromatic amino acid (*aroG*), while the down-regulated proteins were pertinent to the synthesis of alanine (*alr*), arginine (*argF*) and branched-chain amino acid (*ilvE*). The above data implied that eugonic cells enhanced amino acid synthesis in dark conditions so as to support cell growth and proliferation with enough carbon and nitrogen sources. The metatranscriptomic results for the microbial community also suggest that nighttime accumulation of amino acid is an important pathway of cellular nitrogen storage (38). Furthermore, in the stationary phase, cells under the light regimen and dark conditions may require different amino acids as a result of the light-stimulated cellular DOM availability to heterotrophic metabolism (1), and light-enhanced synthesis of glutamate in the stationary phase may be involved in the production of glutathione that protects cells against light stress. Considering leucine, in the exponential phase it was synthesized and converted into components of general metabolism, such as acetoacetate and acetyl-CoA, to participate in active carbohydrate metabolism, and the initiation of the stationary phase led to its degradation in the dark by the detection of down-regulated isovaleryl-CoA dehydrogenase (*ivd*).

**Fig. 8:**
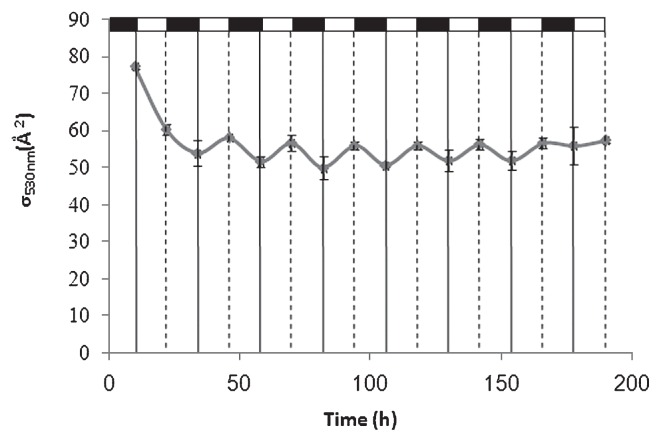
Functional absorption cross-sections (σ) determined by FIRe from whole cell samples after excitation at 530 nm. The top zebra stripe indicates the dark:light (12 h:12 h) cycles of the light regimen. The experiment was performed three times, and the means ± standard errors of the mean are shown.

The response of photosynthetic units in *R. litoralis* OCh149 to the light regimen was directly followed by fluorescence induction and relaxation (FIRe) measurements that generated variable BChl *a* fluorescence transients (26). Analysis of the induction kinetics provided information on the functional absorption cross-section (σ) size of the photosynthetic unit (reaction center and light-harvesting systems) (24). The time-series detection of the value at 12 h intervals showed that it displayed a mild increase under light conditions, but under dark incubation it decreased reversibly at the end of the dark period and reached an elevated level after 12 hours of light (Fig. 8). This indicated that low light intensity stimulated the synthesis of the light-harvesting complexes. In some purple photosynthetic bacteria, such as *Rhodopseudomonas palustris* and *Rhodospirillum photometricum*, more antenna rings developed under low-light conditions (43, 44). In addition, proteins related to stress and chemotaxis were up-regulated in the exponential phase. This phenomenon may be considered as the response of active cells to light stimulation by the functioning of PA proteins.

In summary, light can regulate *R. litoralis* OCh149 photoheterotrophic metabolism by inhibiting its carbohydrate metabolism and stimulating the formation of a functional absorption cross-section and cellular chemotaxis. In addition, in different growth phases, light can enhance different types of amino acid biosynthesis.

## Conclusions

Proteomic analysis of *R. litoralis* OCh149 in the present study provided evidence for an adaptive mechanism in AAPB to stressful environments and the role of light in regulating photoheterotrophic metabolism. The significant proportion of transporters in *R. litoralis* OCh149 demonstrated its effective strategy for rapid carbon/nutrient acquisition; the observed PHA storage and re-utilization showed an intracellular carbon regulation mechanism of *R. litoralis* OCh149 in accordance with extracellular carbon availability. The recorded high proportion of periplasmic glutamate/glutamine/asparate/asparagines-binding proteins in the total transporter spectra along with their corresponding metabolism pathways confirmed the preference for glutamate by *R. litoralis* OCh149, suggesting bacterial selective use of carbon sources. Light-induced variations in carbohydrate and amino acid metabolism indicated that light plays a role in the regulation of heterotrophic metabolism in AAPB. These findings indicate that proteomics allow the analysis of comprehensive mechanisms such as metabolic adaptations to environmental conditions. Further proteomic study of more AAPB type strains will provide insights into photoheterotrophic processes mediating carbon and energy cycles in the ocean.

## Supplementary Material



## Figures and Tables

**Table 1:** Survey of key genes in glucose metabolic pathways from 39 *Roseobacter* clade genomes^a^

Genome	EMP	ED	Oxidative PP	Non-oxidative PP	

	*pfk*	*eda*	*pgd*	*tkt*	*tal*
*Citreicella* sp. SE45	+	+	+	+	+
*Oceanicola granulosus* HTCC2516	+	+	+	+	+
*Pelagibaca bermudensis* HTCC2601	+	+		+	+
*Dinoroseobacter shibae* DFL12	+	+		+	+
*Maritimibacter alkaliphilus* HTCC2654	+	+		+	+
*Sagittula stellata* E-37	+	+		+	+

*Roseibium sp* TrichSKD4		+	+	+	+
*Jannaschia* sp. CCS1		+	+	+	+
*Oceanibulbus indolifex* HEL-45		+	+	+	+
*Octadecabacter antarcticus* 238		+	+	+	+
*Octadecabacter antarcticus* 307		+	+	+	+
*Ketogulonicigenium vulgare* Y25		+	+	+	+
*Rhodobacterales bacterium* HTCC2150		+	+	+	+
*Roseobacter* sp. CCS2		+	+	+	+
*Roseobacter sp.* GAI101		+	+	+	+
*Roseovarius* sp. TM1035		+	+	+	+
*Sulfitobacter* sp. EE-36		+	+	+	+
*Sulfitobacter sp.* NAS-14.1		+	+	+	+
*Thalassiobium* sp. R2A62		+	+	+	+

*Rhodobacterales bacterium* Y4I		+		+	+
*Ruegeria pomeroyi* DSS-3		+		+	+
*Ruegeria* sp. TM1040		+		+	+
*Roseobacter denitrificans* OCh 114		+		+	+
*Roseobacter litoralis* OCh149		+		+	+
*Loktanella vestfoldensis* SKA53		+		+	+
*Oceanicola batsensis* HTCC2597		+		+	+
*Phaeobacter gallaeciensis* BS107		+		+	+
*Phaeobacter gallaeciensis* 2.10		+		+	+
*Rhodobacterales bacterium* HTCC2083		+		+	+
*Roseobacter* sp. AzwK-3b		+		+	+
*Roseobacter* sp. MED193		+		+	+
*Roseobacter* sp. SK209-2-6		+		+	+
*Ruegeria lacuscaerulensis* ITI-1157		+		+	+
*Ruegeria* sp. TrichCH4B		+		+	+
*Ruegeria* sp. R11		+		+	+
*Rhodobacterales bacterium* KLH11		+		+	+

*Rhodobacterales bacterium* HTCC2255		+		+	

*Roseovarius nubinhibens* ISM			+	+	+
*Roseovarius* sp. 217			+	+	+

a + indicates the presence of a homolog with an E value ≤10^−40^ and amino acid percent sequence identity ≥40%.

**Table 2:** Proteins up- or down-regulated in exponential phase

No.^a^	Accession No.	Gene	Protein Name	MW kDa	PI	Pep.Hits	Protein Score	Fold Change^b^
**Carbohydrate Metabolism**								
EU9	gi|163732692		alcohol dehydrogenase, zinc-containing, putative	35.40	5.05	13	804	−2.33
EU16	gi|163732764	*acsA*	acetyl-coenzyme A synthetase	69.53	5.37	25	682	1.58
EU21	gi|163732974	*tme*	malic enzyme	80.96	5.58	26	469	L
ED17	gi|163731504	*eda*	KHG/KDPG aldolase	21.62	4.55	6	131	−5
ED34	gi|163731505	*frk*	fructokinase	32.66	4.62	8	540	−6.66
ED43	gi|163734895	*tpiA*	triosephosphate isomerase	28.06	4.86	14	360	−4.55
ED60	gi|163731508	*glgC*	glucose-1-phosphate adenylyltransferase	47.01	5.46	30	983	−2.44
ED63	gi|163734455	*coxG*	carbon monoxide dehydrogenase G protein, putative	15.85	5.50	12	549	−1.53
ED79	gi|163732070	*mvaB*	hydroxymethylglutaryl-CoA lyase	30.53	4.96	16	644	−33.3
ED61	gi|163735981		UDP-glucose/GDP-mannose dehydrogenase, putative	48.88	5.44	7	169	−5.88
ED36	gi|163732615		PfkB family kinase, putative	35.03	4.66	15	531	−3.45
**Photosynthesis and Oxidative Phosphorylation**								
ED54	gi|163736059	*cobT*	nicotinate-nucleotide--dimethylbenzimidazole phosphoribosyltransferase	20.54	4.59	3	216	−2.44
ED72	gi|110677947	*petA*	ubiquinol-cytochrome c reductase, iron-sulfur subunit	20.10	4.55	8	529	−1.89
**Fatty acid Metabolism**								
ED28	gi|163731533	*accB*	acetyl-CoA carboxylase, biotin carboxyl carrier protein, putative	16.94	4.70	4	124	−7.15
**Amino acid Metabolism**								
EU10	gi|163734687	*hisD*	histidinol dehydrogenase	51.40	5.46	14	411	2.20
ED39	gi|163732719	*hisD*	histidinol dehydrogenase	45.93	4.78	17	604	−2.08
EU18	gi|163732684	*ald*	alanine dehydrogenase	38.74	5.71	19	722	2.23
ED16	gi|163732180	*hisA*	1-(5-phosphoribosyl)-5-[(5-phosphoribosylamino)methyli deneamino] imidazole-4-carboxamide isomerase	24.80	4.55	14	822	−2.63
ED35	gi|163735980	*fmdA*	formamidase, putative	33.38	4.76	6	168	−3.45
ED32	gi|163731988	*trpC*	indole-3-glycerol phosphate synthase	29.42	4.71	17	503	−2.22
ED41	gi|163733282	*leuB*	3-isopropylmalate dehydrogenase	39.96	4.63	10	263	−2.78
ED42	gi|163733060	*arcB*	ornithine cyclodeaminase, putative	33.21	4.84	17	782	−5
ED45	gi|163734587	*aspB*	aspartate aminotransferase	43.35	4.86	17	494	−3.03
ED76	gi|163731559	*aspB*	aspartate aminotransferase, putative	39.31	4.67	16	773	−1.72
ED50	gi|163733195	*serB*	phosphoserine phosphatase, putative	30.72	4.90	7	128	−2.04
ED51	gi|163731381	*mmsA*	methylmalonate-semialdehyde dehydrogenase	53.98	5.15	24	684	−2.22
ED56	gi|163735924	*paaK*	phenylacetate-CoA ligase, putative	44.68	5.29	13	349	−2.04
ED65	gi|163731568	*ilvH*	acetolactate synthase 3 small subunit	20.13	5.77	8	277	−2.78
ED67	gi|163733251	*serA*	D-3-phosphoglycerate dehydrogenase	56.27	5.67	11	268	−2.63
ED68	gi|163733251	*serA*	D-3-phosphoglycerate dehydrogenase	56.27	5.67	23	834	−2.86
ED85	gi|163733589	*ipuC*	gamma-glutamylisopropylamide synthetase, putative	51.04	5.28	20	346	−1.92
ED89	gi|163731868	*aroG*	phospho-2-dehydro-3-deoxyheptonate aldolase	51.19	5.82	27	1,080	−1.82
ED90	gi|163733560	*thrC*	threonine synthase	50.56	5.84	22	852	−2.38
ED93	gi|163731356	*cysE*	serine acetyltransferase	29.35	6.10	22	965	−1.96
**Purine/Pyrimidine Metabolism**								
ED48	gi|163734274		polyribonucleotide nucleotidyltransferase	76.75	4.88	30	1,090	−2.33
ED77	gi|163732267		thioredoxin-disulfide reductase	35.16	4.90	8	174	−1.75
ED91	gi|163731781	*guaB*	inosine-5′-monophosphate dehydrogenase	50.75	5.83	24	736	−1.85
ED69	gi|163731781	*guaB*	inosine-5′-monophosphate dehydrogenase	50.75	5.83	26	984	−2.08
**Transcription and Translation**								
EU4	gi|163734097	*npdA*	NAD-dependent deacetylase	26.53	5.04	7	367	2.07
EU5	gi|163732886	*tufA*	elongation factor Tu	42.84	4.90	19	802	5.41
EU7	gi|110679879	*rpsB*	30S ribosomal protein S2	28.19	5.02	10	448	2.05
EU19	gi|163733470	*proS*	prolyl-tRNA synthetase	51.03	5.71	27	696	L
ED44	gi|163734600		endonuclease, putative	39.54	4.76	18	898	−2.13
ED81	gi|163735131	*rnd*	ribonuclease D	43.88	5.39	16	298	−1.79
ED86	gi|163735041	*thrS*	threonyl-tRNA synthetase	73.82	5.53	35	977	−1.92
ED87	gi|163732970	*rph*	ribonuclease PH	25.55	5.55	9	514	−5.26
ED96	gi|163735421	*ppsR*	transcriptional regulator PpsR	51.50	5.68	27	850	D
**Transporters**								
ED2	gi|163735254		amino-acid ABC transporter, periplasmic binding protein	30.06	4.37	11	355	−3.13
ED4	gi|163735254		amino-acid ABC transporter, periplasmic binding protein	30.06	4.37	16	558	−3.12
ED22	gi|163731684		branched-chain amino acid ABC transporter, amino acid-binding protein	42.35	4.55	16	715	−2
ED3	gi|163735168		putative extracellular solute-binding protein	29.86	4.48	8	365	−2.5
ED25	gi|163733639		bacterial extracellular solute-binding protein, family 5	57.44	4.56	19	752	−3.23
ED7	gi|163735575		ferric iron ABC transporter, periplasmic ferric iron-binding protein	36.51	4.31	8	356	−2.00
ED9	gi|163731673		ABC transporter, binding protein	39.77	4.40	11	644	−2.28
ED11	gi|163731860	*pstS*	phosphate ABC transporter, periplasmic phosphate-binding protein	35.56	4.19	9	801	−2.78
ED12	gi|163733174	*potF*	putrescine ABC transporter, periplasmic putrescine-binding protein	39.57	4.30	8	362	−3.45
ED20	gi|163735933		C4-dicarboxylate binding protein, putative	37.17	4.65	15	680	−4.76
ED21	gi|163735933		C4-dicarboxylate binding protein, putative	37.17	4.65	16	660	−2.44
ED23	gi|163735737		TRAP dicarboxylate transporter, DctP subunit, putative	36.53	4.57	8	141	−5
ED38	gi|163734761		sugar ABC transporter, periplasmic binding protein, putative	47.91	4.72	17	885	−2.22
ED82	gi|163735383		spermidine/putrescine ABC transporter, ATP-binding protein	40.37	5.30	14	175	−33.3
**Stress Proteins**								
EU8	gi|163735915	*clpB*	ATP-dependent Clp protease, ATP-binding subunit ClpB	95.34	5.03	43	937	2.49
EU15	gi|163731525	*clpP*	Clp protease, putative	23.06	5.27	10	343	2.00
EU11	gi|163734797	*htpG*	heat shock protein 90	73.10	5.26	11	76	L
EU17	gi|163732530		heat shock protein, Hsp20 family	17.52	5.66	15	580	2.14
EU13	gi|163732530		heat shock protein, Hsp20 family	17.52	5.66	10	299	L
EU23	gi|163734278		antioxidant, AhpC/Tsa family, putative	23.58	4.95	11	209	50
ED24	gi|163733305		serine protease DO-like precursor, putative	51.46	4.50	25	749	−4.17
ED84	gi|163735794	*pepA*	leucyl aminopeptidase	51.59	5.33	21	1,090	−1.79
**Chemotaxis**								
EU24	gi|110680249	*cheY*	chemotaxis protein CheY, putative	13.11	5.07	9	351	L
**Others**								
ED71	gi|163732956		protein-export protein SecB, putative	18.92	4.70	11	436	−2.16
ED37	gi|163732221	*dacC*	D-alanyl-D-alanine carboxypeptidase	41.66	4.71	17	685	−2.33
EU1	gi|163734456		arylsulfatase	58.27	4.73	11	184	2.95
EU6	gi|163734456		arylsulfatase	58.27	4.73	20	936	2.01
EU2	gi|163735358		sulfatase, putative	60.10	4.78	21	720	2.38
EU20	gi|163734500	*pta*	phosphate acetyltransferase	82.30	5.55	29	554	3.42
EU22	gi|115345666		bifunctional sulfate adenylyltransferase subunit 1/adenylylsulfate kinase protein	64.39	5.67	23	691	2.48
ED1	gi|163731557		outer membrane protein, putative	27.77	4.44	14	803	−2.33
ED5	gi|163732591		VacJ-like lipoprotein, putative	26.01	4.44	16	679	−3.45
ED8	gi|163732359	*ggt*	gamma-glutamyltranspeptidase	61.18	4.52	8	202	−8.33
ED14	gi|163735712		YceI-like family protein	20.76	4.58	7	305	−3.23
ED26	gi|115345661		TPR repeat-containing protein	88.21	4.37	14	300	−2.13
ED30	gi|163733017	*cysQ*	3′(2′),5′-bisphosphate nucleotidase	28.71	4.62	17	850	−2.13
ED49	gi|163734774		monooxygenase protein, putative	67.94	4.95	21	508	−3.57
ED52	gi|163732119		metallopeptidase, family M24, putative	64.79	5.12	22	454	−5.56
ED53	gi|163731615		putative acetyltransferase	24.09	5.33	11	179	−2.33
ED57	gi|163731748	*acy*	penicillin acylase, putative	91.03	5.13	18	459	−2.27
ED58	gi|163734508		AMP-binding domain protein	58.70	5.23	19	702	−2.13
ED62	gi|163733176		putative aminotransferase	50.48	5.46	9	121	−2.38
ED66	gi|163733218		zinc-binding dehydrogenase	36.77	5.59	13	383	−2.00
ED75	gi|163732812		immunogenic protein, putative	34.13	4.87	13	515	−2.86
ED83	gi|163735942		amidohydrolase family protein	41.94	5.27	18	528	−4.17
ED88	gi|163735799	*pdxA*	4-hydroxythreonine-4-phosphate dehydrogenase	34.31	5.77	8	285	−2.84
ED92	gi|163732023		oxidoreductase, putative	22.12	5.93	10	356	−2.07
ED94	gi|163735137		metallo-beta-lactamase family protein, putative	33.56	6.06	9	252	−3.13
**Hypothetical Proteins**								
EU25	gi|163735582		unkown protein RLO149_07769	17.02	5.16	4	117	7.93
ED13	gi|163735835		unkown protein RLO149_00770	43.35	4.27	10	369	−2.44
ED19	gi|163731610		unkown protein RLO149_19939	21.62	4.59	8	313	−4.76
ED31	gi|163732009		unkown protein RLO149_01122	33.60	4.69	12	336	−2.70
ED33	gi|163732240		unkown protein RLO149_02277	36.23	5.59	13	316	−3.45
ED46	gi|163734706		unkown protein RLO149_14803	45.06	4.85	11	198	−3.33
ED55	gi|163731979		unkown protein RLO149_00972	42.28	5.46	11	316	−2.33
ED59	gi|163732720		unkown protein RLO149_12800	18.17	5.37	17	592	−2.56
ED74	gi|163732284		unkown protein RLO149_02497	31.69	4.88	11	251	−1.89
ED80	gi|163735585		unkown protein RLO149_07784	34.70	5.20	11	426	−33.3
ED95	gi|163732428		unkown protein RLO149_03217	28.68	4.86	13	500	−1.72

a EU, up-regulated in light regimen in exponential phase; ED, down-regulated in light regimen in exponential phase

b L, only expressed under light regimen conditions; D, only expressed under dark conditions

**Table 3:** Proteins up- or down-regulated in stationary phase

No.^a^	Accession No.	Gene	Protein Name	MWkDa	PI	Pep. Hits	Protein Score	Fold Change^b^
**Carbohydrate Metabolism**								
SU10	gi|163731361	*pgk*	phosphoglycerate kinase	41.43	5.09	15	466	2.34
SU11	gi|163734512	*phaA*	acetyl-CoA acetyltransferase	43.23	5.05	15	477	4.60
SU12	gi|115345672	*xanB*	mannose-1-phosphate guanylyltransferase	50.85	5.20	19	954	5.24
SU21	gi|163734174	*IpdA*	dihydrolipoamide dehydrogenase	49.37	6.15	14	357	2.33
SD8	gi|115345653	*rfbA*	glucose-1-phosphate thymidylyltransferase	31.67	4.90	4	99	D
SD13	gi|163735839	*nagZ*	beta-hexosaminidase, putative	36.13	5.19	21	917	−11.11
SD14	gi|163733474		malate/L-lactate dehydrogenase, putative	37.29	6.08	3	223	−7.69
SD24	gi|163731409	*tpiA*	triosephosphate isomerase	25.60	4.92	7	359	D
SD27	gi|163734747	*fucU*	fucose operon FucU protein, putative	16.28	4.86	13	1,010	−3.85
SD33	gi|163731776	*pgl*	6-phosphogluconolactonase	23.75	5.06	8	503	−2.08
SD38	gi|163733019	*gtaB*	UTP--glucose-1-phosphate uridylyltransferase	33.21	5.22	18	525	−4.17
SD42	gi|163733025	*kdsD*	arabinose 5-phosphate isomerase	33.94	5.74	11	533	−2.63
**Photosynthesis and Oxidative Phosphorylation**								
SU13	gi|163732297	*atpA*	F0F1 ATP synthase subunit alpha	54.68	5.26	23	633	4.03
SD22	gi|163732324		electron transfer flavoprotein-ubiquinone oxidoreductase, putative	60.01	5.59	26	1,120	D
SD29	gi|163734669	*cobH*	precorrin-8X methylmutase	22.45	5.08	5	254	−3.70
**Fatty acid Metabolism**								
SD16	gi|163731882	*lpxD*	UDP-3-O-3-hydroxymyristoyl glucosamine N-acyltransferase	38.27	5.52	19	727	−6.67
SD2	gi|163734510	*fadJ*	fatty acid oxidation complex alpha subunit, putative	78.48	5.61	32	815	−2.21
SD40	gi|163734510	*fadJ*	fatty acid oxidation complex alpha subunit, putative	78.48	5.61	33	1,080	−2.33
**Amino acid Metabolism**								
SU2	gi|163731868	*aroG*	phospho-2-dehydro-3-deoxyheptonate aldolase	51.19	5.82	16	464	L
SU19	gi|163733991	*gill*	glutamate dehydrogenase	51.48	5.91	26	569	2.64
SU20	gi|163733991	*gill*	glutamate dehydrogenase	51.48	5.91	15	445	6.09
SU22	gi|163732180	*hisA*	1-(5-phosphoribosyl)-5-[(5-phosphoribosylamino)methyli deneamino] imidazole-4-carboxamide isomerase	24.80	4.55	6	157	L
SD6	gi|163734513	*gst*	glutathione S-transferase, putative	23.79	5.14	16	659	−25
SD20	gi|110678669	*fahd*	fumarylacetoacetate hydrolase, putative	24.61	5.66	5	115	−8.33
SD21	gi|163734433	*ilvE*	branched-chain amino acid aminotransferase	33.73	5.84	18	816	−2.08
SD30	gi|163732015	*alr*	alanine racemase	36.75	4.99	17	1,040	−2.00
SD34	gi|163732077	*ivd*	isovaleryl-CoA dehydrogenase	41.89	5.09	16	471	−2.56
SD35	gi|163731381	*mmsA*	methylmalonate-semialdehyde dehydrogenase	53.98	5.15	19	1,020	−3.03
SD39	gi|163734103	*argF*	ornithine carbamoyltransferase	34.26	5.41	15	747	−2.44
**Purine/Pyrimidine Metabolism**								
SU17	gi|163735199	*hydA*	dihydropyrimidinase	53.12	5.51	20	391	9.68
**Transcription and Translation**								
SU1	gi|163732886	*tufA*	elongation factor Tu	42.84	4.90	17	1,020	L
SU9	gi|163733850	*chvI*	DNA-binding response regulator ChvI, putative	26.68	4.96	17	897	2.05
SD18	gi|163733026		3′-5′ exonuclease family protein, putative	22.98	5.70	13	669	−50
SD32	gi|163732327	*greA*	transcription elongation factor GreA	17.14	5.03	5	133	−2.18
**Transporters**								
SU5	gi|163731673		ABC transporter, binding protein	39.77	4.40	15	769	2.64
SU6	gi|163732488		ABC transporter, ATP-binding protein	21.94	4.65	10	528	2.57
SD4	gi|163735372		sugar ABC transporter, substrate-binding protein, putative	36.49	4.48	9	674	D
SD10	gi|163733177		polyamine ABC transporter, ATP-binding protein, putative	41.39	5.08	20	694	−12.5
**Stress Proteins**								
SD11	gi|163734560	*phoH*	phosphate starvation inducible protein, putative	36.90	5.09	14	396	−10.72
**Others**								
SU4	gi|163731557		outer membrane protein, putative	27.77	4.44	14	524	7.33
SU14	gi|163731748	*acy*	penicillin acylase, putative	91.03	5.13	23	604	9.58
SU15	gi|163733001	*ubiG*	3-demethylubiquinone-9 3-methyltransferase	27.74	5.41	11	387	1.05
SU18	gi|163732359	*ggt*	gamma-glutamyltranspeptidase	61.18	4.52	10	739	2.30
SD3	gi|163735850		phospholipase/carboxylesterase family protein	23.59	4.38	5	153	D
SD7	gi|163736040		oxidoreductase, putative	29.79	5.07	15	683	−33.3
SD9	gi|163734732		oxidoreductase, putative	40.46	5.28	25	914	−33.3
SD12	gi|163732119		metallopeptidase, family M24, putative	64.79	5.12	21	494	−5.88
SD17	gi|163733176		putative aminotransferase	50.48	5.46	10	329	−11.11
SD19	gi|163735045		hydrolase, putative	26.05	5.73	11	394	−20
SD26	gi|163731362		peptidyl-prolyl cis-trans isomerase, cyclophilin-type, putative	18.17	4.74	7	566	−2.08
SD28	gi|163734126		NifU-like domain protein	20.21	4.92	7	356	−2.22
SD36	gi|163734683		decarboxylase, putative	18.74	5.16	4	237	−2.33
SD43	gi|163732693	*qor*	quinone oxidoreductase	34.36	5.88	10	534	−2.33
**Unkown Proteins**								
SU3	gi|163732454		unkown protein RLO149_03347	19.88	4.66	10	732	3.02
SU7	gi|163731750		unkown protein RLO149_20639	22.84	6.29	3	193	2.54
SU8	gi|163734708		unkown protein RLO149_14813	27.79	4.85	5	99	4.12
SD15	gi|163732606		unkown protein RLO149_12230	21.07	5.27	11	382	−7.69
SD25	gi|163731436		unkown protein RLO149_19069	32.66	4.45	11	652	−3.45
SD37	gi|163734540		unkown protein RLO149_13973	35.81	5.54	6	179	−2.63
SD41	gi|163735269		unkown protein RLO149_06937	25.45	5.66	23	983	−2.27

a SU, up-regulated in light regimen in stationary phase; SD, down-regulated in light regimen in stationary phase

b L, only expressed under light regimen conditions; D, only expressed under dark conditions
