# Supplementary material for: Proteomic Responses of Roseobacter litoralis OCh149 to Starvation and Light Regimen
Source: Microbes Environ. 2012 Oct 5;27(4):430–42. doi: 10.1264/jsme2.ME12029 (PMC4103551; doi:10.1264/jsme2.ME12029)
Supplement: Supplementary file 1 [file 27_430_s1.pdf]

Table S1. Proteins detected at the stationary phase by LC-MS/MS

Accession No.	Gene	Protein	No. of Unique Peptides	Spectral Count	Expression[a]
**Carbon metabolism**					
ZP_02139222	*zwf*	glucose-6-phosphate- 1-dehydrogenase	2	2	D
ZP_02139224	*pgi*	glucose-6-phosphate isomerase	2	3	D
ZP_02142219	*xylA*	xylose isomerase	3	5	Both
ZP_02142552	*fba*	fructose-bisphosphate aldolase	5	15	Both
ZP_02138949	*gap*	glyceraldehyde-3-phosphate dehydrogenase	6	16	Both
ZP_02143248	*eno*	enolase	4	20	Both
ZP_02138950	*edd*	phosphogluconate dehydratase	5	7	Both
ZP_02138834	*tkt*	transketolase	4	8	Both
ZP_02139737	*prs*	ribose-phosphate pyrophosphokinase putative	5	12	Both
ZP_02140102	*pyk*	pyruvate kinase	1	3	L
ZP_02139594	*pyc*	pyruvate carboxylase	5	6	D
ZP_02141600	*mdh*	malate dehydrogenase	12	42	Both
ZP_02139440	*gltA*	citrate synthase	7	18	Both
ZP_02139391	*acn*	aconitate hydratase	4	8	D
ZP_02139240	*icd*	isocitrate dehydrogenase	3	4	D
ZP_02141063	*icd*	isocitrate dehydrogenase, NADP-dependent	6	10	D
ZP_02141608	*sucA*	alpha-ketoglutarate dehydrogenase	9	32	Both
ZP_02141609	*aceF*	dihydrolipoamide acetyltransferase	8	29	Both
ZP_02141615	*dld*	dihydrolipoamide dehydrogenase	5	15	Both
ZP_02138821	*dld*	dihydrolipoamide dehydrogenase	2	5	D
ZP_02138806	*pdhA*	pyruvate dehydrogenase complex, E1 component, alpha subunit	1	3	D
ZP_02140209	*acs*	acetyl-coenzyme A synthetase	2	3	D
ZP_02141602	*sucC*	succinyl-CoA synthetase, beta subunit	9	28	Both
ZP_02141606	*sucD*	succinyl-CoA synthetase, alpha subunit	8	32	Both
ZP_02139761	*phaB*	acetoacetyl-CoA reductase, putative	5	11	L
ZP_02139760	*phaA*	acetyl-CoA acetyltransferase	7	21	Both
ZP_02141951	*phaA*	acetyl-CoA acetyltransferase	2	3	D
ZP_02142486	*phaR*	polyhydroxyalkanoate synthesis repressor	1	3	D
ZP_02141589	*sdhA*	succinate dehydrogenase flavoprotein subunit	2	2	D
ZP_02142549	*fumC*	fumarate hydratase	5	8	Both
ZP_02141689	*aldh*	aldehyde dehydrogenase family protein, putative	2	3	L
ZP_02138844	*coxM*	carbon monoxide dehydrogenase, medium subunit	3	8	Both
ZP_02139547	*glcB*	malate synthase G	8	19	Both
ZP_02143293	*mxaF*	methanol dehydrogenase, large subunit	4	11	Both
ZP_02139511	*nuoC*	NADH dehydrogenase subunit C	3	7	Both
ZP_02139499	*nuoG*	NADH dehydrogenase subunit G	2	2	D
**Photosynthesis and oxidative phosphorylation**					
ZP_02142898	*pufC*	photosynthetic reaction center cytochrome c subunit	3	9	Both
ZP_02141769		NADH-ubiquinone oxidoreductase, putative	1	3	L
ZP_02142870	*cycA*	cytochrome c-551	2	11	Both
ZP_02141863	*petA*	ubiquinol-cytochrome c reductase, iron-sulfur subunit	1	3	L
ZP_02141343	*atpF*	ATP synthase F0, B subunit	4	10	L
ZP_02141344	*atpX*	ATP synthase F0, B' subunit	4	17	Both
ZP_02139743	*atpA*	F0F1 ATP synthase subunit alpha	16	66	Both
ZP_02139741	*atpD*	F0F1 ATP synthase subunit beta	14	57	Both
ZP_02139742	*atpG*	F0F1 ATP synthase subunit gamma	3	3	L
ZP_02142877	*bchO*	magnesium-chelatase 38 kDa subunit	3	7	Both
ZP_02141383	*hemC*	porphobilinogen deaminase	6	12	Both
ZP_02139198	*hemB*	delta-aminolevulinic acid dehydratase	3	4	D
ZP_02141382	*hemE*	uroporphyrinogen decarboxylase	3	3	L
ZP_02142851	*bchP*	geranylgeranyl hydrogenase	4	6	D
ZP_02139729	*ppaC*	inorganic pyrophosphatase, manganese-dependent	4	11	Both
**Fatty acid metabolism**					
ZP_02141283	*etfA*	electron transfer flavoprotein, alpha subunit	4	14	Both
ZP_02141282	*etfB*	electron transfer flavoprotein, beta subunit	7	21	Both
ZP_02140904	*mut*	methylmalonyl-CoA mutase	5	6	Both
ZP_02139206	*bdh*	D-beta-hydroxybutyrate dehydrogenase	4	11	Both
ZP_02141996	*fabA*	3-hydroxydecanoyl-ACP dehydratase	2	6	L
ZP_02138783	*fabB*	3-oxoacyl-(acyl carrier protein) synthase	1	3	L
ZP_02141995	*fabB*	3-oxoacyl-(acyl-carrier-protein) synthase	3	8	Both
ZP_02140699	*fadA*	beta-ketothiolase	2	9	Both
**Amino acid metabolism**					
ZP_02139527	*glmS*	D-fructose-6-phosphate amidotransferase	2	2	D
ZP_02140099	*dat*	D-alanine aminotransferase	8	23	Both
ZP_02140129	*ald*	alanine dehydrogenase	5	12	Both
ZP_02140030	*aspC*	aspartate aminotransferase	2	2	D
ZP_02142026	*aspC*	aspartate aminotransferase	3	6	Both
ZP_02140735	*adss*	adenylosuccinate synthetase	2	4	D
ZP_02140422	*argG*	argininosuccinate synthase	2	3	D
ZP_02141432	*glud*	glutamate dehydrogenase	13	35	Both
ZP_02139377	*glnA*	glutamine synthetase, type I	7	15	Both

Accession No.	Gene	Protein	No. of Unique Peptides	Spectral Count	Expression[a]
ZP_02140695	*serA*	D-3-phosphoglycerate dehydrogenase	6	7	Both
ZP_02138803	*cysE*	serine acetyltransferase	3	11	Both
ZP_02139899	*metB*	O-acetylhomoserine sulfhydrylase, probable	6	14	Both
ZP_02138887	*cysK*	O-acetylserine sulfhydrylase	5	8	Both
ZP_02140777	*ilvC*	ketol-acid reductoisomerase	4	6	Both
ZP_02140495	*ilvD*	dihydroxy-acid dehydratase	4	14	Both
ZP_02141873	*ilvE*	branched-chain amino acid aminotransferase	2	4	L
ZP_02142012	*trpB*	tryptophan synthase subunit beta	2	2	L
ZP_02142022	*trpA*	tryptophan synthase, alpha subunit	2	8	Both
ZP_02140521	*asd*	aspartate-semialdehyde dehydrogenase	2	2	L
ZP_02140022	*aldh*	succinate-semialdehyde dehydrogenase	2	3	D
ZP_02138804	*dbt*	branched-chain alpha-keto acid dehydrogenase E2 subunit	4	15	Both
ZP_02142003	*metK*	S-adenosylmethionine synthase	3	4	D
ZP_02140697	*kbl*	2-amino-3-ketobutyrate coenzyme A ligase	7	19	Both
ZP_02140726	*leuB*	3-isopropylmalate dehydrogenase	4	7	D
ZP_02140069	*dapA*	dihydrodipicolinate synthase	2	8	Both
ZP_02140763	*metZ*	O-succinylhomoserine sulfhydrylase	2	3	D
ZP_02143223	*lap*	leucyl aminopeptidase	9	17	Both
ZP_02140378	*ahcY*	S-adenosyl-L-homocysteine hydrolase	2	4	D
ZP_02143463	*hpcE*	2-hydroxyhepta-2,4-diene-1,7-dioate isomerase, putative	4	13	Both
**Purine/pyrimidine metabolism**					
ZP_02141744	*trx*	thioredoxin, putative	1	3	L
ZP_02143344	*pyrF*	orotidine 5'-phosphate decarboxylase	1	3	L
ZP_02142635	*hydA*	dihydropyrimidinase	1	3	D
ZP_02139228	*guaB*	inosine-5'-monophosphate dehydrogenase	5	14	Both
ZP_02139386	*purA*	adenylosuccinate lyase	1	4	L
ZP_02143216	*ndk*	nucleoside diphosphate kinase	2	5	L
ZP_02140707	*dnaN*	DNA polymerase III subunit beta	8	27	Both
ZP_02141714	*pnp*	polyribonucleotide nucleotidyltransferase	7	14	Both
ZP_02141694	*purH*	bifunctional phosphoribosylaminoimidazolecarboxamide formyltransferase/IMP cyclohydrolase	2	6	Both
ZP_02141965		putative 5-nucleotidase	2	4	Both
**Transcription and translation**					
ZP_02139347	*ctrA*	transcriptional regulator CtrA	3	7	Both
ZP_02140320	*nusG*	transcription antitermination protein NusG	2	3	D
ZP_02140406	*rho*	transcription termination factor Rho	4	7	Both
ZP_02140449	*nusA*	transcription elongation factor NusA	2	2	L
ZP_02140784	*gntR*	transcriptional regulator, GntR family, putative	2	2	D
ZP_02140451	*infB*	translation initiation factor IF-2	2	2	L
ZP_02138847	*infC*	translation initiation factor IF-3	2	8	Both
ZP_02140330	*fusA*	elongation factor G	12	25	Both
ZP_02139299	*tsf*	elongation factor Ts	3	11	Both
ZP_02140331	*tuf*	elongation factor Tu	14	45	Both
ZP_02142008	*rpsA*	30S ribosomal protein S1	2	3	D
ZP_02141271	*rpsJ*	30S ribosomal protein S10	2	7	Both
ZP_02141226	*rpsK*	30S ribosomal protein S11	4	7	Both
ZP_02141227	*rpsM*	30S ribosomal protein S13	7	16	Both
ZP_02139298	*rpsB*	30S ribosomal protein S2	3	5	Both
ZP_02141261	*rpsC*	30S ribosomal protein S3	3	7	Both
ZP_02139757	*rpsD*	30S ribosomal protein S4	7	18	Both
ZP_02138774	*rpsF*	30S ribosomal protein S6	2	3	D
ZP_02140329	*rpsG*	30S ribosomal protein S7	3	13	Both
ZP_02141242	*rpsH*	30S ribosomal protein S8	2	10	Both
ZP_02140322	*rplA*	50S ribosomal protein L1	1	3	D
ZP_02140321	*rplK*	50S ribosomal protein L11	1	3	L
ZP_02141246	*rplN*	50S ribosomal protein L14	1	3	L
ZP_02141260	*rplP*	50S ribosomal protein L16	2	7	Both
ZP_02141224	*rplQ*	50S ribosomal protein L17	1	3	L
ZP_02141240	*rplR*	50S ribosomal protein L18	3	10	Both
ZP_02141361	*rplS*	50S ribosomal protein L19	2	10	Both
ZP_02140106	*rplT*	50S ribosomal protein L20	2	4	D
ZP_02139285	*rplU*	50S ribosomal protein L21/unknown domain fusion protein	4	14	Both
ZP_02141262	*rplV*	50S ribosomal protein L22	2	3	D
ZP_02141268	*rplW*	50S ribosomal protein L23	1	3	L
ZP_02139286	*rpmA*	50S ribosomal protein L27	1	3	D
ZP_02141238	*rpmD*	50S ribosomal protein L30	1	3	L
ZP_02141269	*rplD*	50S ribosomal protein L4	2	2	L
ZP_02141241	*rplF*	50S ribosomal protein L6	2	3	D
ZP_02138772	*rplI*	50S ribosomal protein L9	5	13	Both
ZP_02141233	*rplO*	50Sribosomal protein L15	3	5	Both
ZP_02141264	*rplB*	50Sribosomal protein L2	4	13	Both
ZP_02141541	*rpmB*	50Sribosomal protein L28	1	3	D
ZP_02140311	*hisS*	histidyl-tRNA synthetase	2	2	L
ZP_02141225	*rpoA*	DNA-directed RNA polymerase subunit alpha	6	17	Both

Accession No.	Gene	Protein	No. of Unique Peptides	Spectral Count	Expression[a]
ZP_02140325	*rpoB*	DNA-directed RNA polymerase subunit beta	10	13	Both
ZP_02140326	*rpoC*	DNA-directed RNA polymerase subunit beta'	7	20	Both
ZP_02139949	*hup*	DNA-binding protein HU	3	21	Both
ZP_02141747	*mtrA*	DNA-binding response regulator, putative	1	3	L
ZP_02142009	*ihfB*	integration host factor subunit beta	2	3	D
ZP_02143263	*ihfA*	integration host factor, alpha subunit	3	6	Both
**Stress protein**					
ZP_02140416	*grpE*	GrpE protein HSP-70 cofactor, putative	1	3	L
ZP_02138972	*clpS*	Clp protease, putative	3	10	Both
ZP_02142235	*htpG*	heat shock protein 90	3	6	Both
ZP_02139975	*hspH*	heat shock protein, Hsp20 family	3	18	Both
ZP_02139716		heat shock protein, Hsp20 family, putative	3	10	Both
ZP_02140459	*dnaK*	chaperone protein DnaK, putative	12	44	Both
ZP_02139724	*groEL*	chaperonin GroEL	34	122	Both
ZP_02139725	*groES*	chaperonin, 10 kDa	5	21	Both
ZP_02143343	*clpB*	ATP-dependent Clp protease, ATP-binding subunit ClpB	17	53	Both
ZP_02138876	*lon*	ATP-dependent protease La, putative	2	2	D
ZP_02139249	*sufC*	FeS assembly ATPase SufC	3	8	Both
ZP_02141718		antioxidant, AhpC/Tsa family, putative	2	4	D
ZP_02141447	*sodB*	superoxide dismutase (Fe)	3	7	Both
ZP_02140855	*telA*	tellurite resistance protein TelA	5	10	Both
ZP_02141709		universal stress family protein, putative	2	3	D
ZP_02140300		universal stress family protein, putative	1	3	L
ZP_02141690	*pqqE*	peptidase, M16 family, putative	2	3	D
ZP_02141305	*lepB*	signal peptidase I, putative	2	3	L
**Chemotaxis**					
ZP_02142765	*mcpA*	methyl-accepting chemotaxis protein McpA	13	40	Both
ZP_02142731		methyl-accepting chemotaxis protein, putative	9	13	D
ZP_02141023		methyl-accepting chemotaxis protein, putative	8	13	L
ZP_02138875		chemoreceptor protein, putative	1	3	L
ZP_02138757	*cheA*	chemotaxis protein CheA	2	7	Both
ZP_02138758	*cheA*	chemotaxis protein CheA	2	3	L
ZP_02138756	*cheW*	chemotaxis protein CheW	2	7	Both
**Transporters**					
ZP_02141887		ABC transporter substrate binding protei	4	7	Both
ZP_02142663		ABC transporter, ATP-binding protein, putative	3	3	D
ZP_02142631		ABC transporter, periplasmic	2	7	Both
ZP_02142041		ABC transporter, periplasmic substrate-binding protein, putative	3	6	D
ZP_02142337		ABC transporter, substrate-binding protein, putative	2	10	Both
ZP_02140622		ABC transporter, substrate-binding protein, putative	3	12	Both
ZP_02142689		amino-acid ABC transporter, periplasmic binding protein	2	4	L
ZP_02141504		bacterial extracellular solute-binding protein, family 7	4	10	Both
ZP_02142163		bacterial extracellular solute-binding protein, family 7,putative	2	2	L
ZP_02140148		C4-dicarboxylate transport system, substrate-binding protein, putative	4	7	Both
ZP_02141381		C4-dicarboxylate-binding periplasmic protein	4	8	Both
ZP_02143160		Extracellular ligand-binding receptor	2	12	Both
ZP_02139643		extracellular solute-binding protein, family 1	4	6	Both
ZP_02139683		extracellular solute-binding protein, family 1	1	3	L
ZP_02141083		extracellular solute-binding protein, putative	6	16	Both
ZP_02143007		ferric iron ABC transporter, periplasmic ferric iron-binding protein	9	50	Both
ZP_02141218		glutamate/glutamine/aspartate/asparagine-binding protein	21	230	Both
ZP_02139636		glycerol-3-phosphate ABC transporter, periplasmic binding protein	8	24	Both
ZP_02138889		glycerol-3-phosphate-binding periplasmic protein precursor, putative	6	22	Both
ZP_02143318		ribose ABC transporter, periplasmic binding component, putative	2	6	Both
ZP_02140156		iron ABC transporter iron-binding protein, putative	2	2	L
ZP_02139537		peptide ABC transporter, periplasmic substrate-binding protein	4	6	Both
ZP_02139021		periplasmic mannitol-binding protein, putative	5	12	Both
ZP_02139307		phosphate ABC transporter, periplasmic phosphate-binding protein	1	3	L
ZP_02140153		phosphonate ABC transporter, periplasmic binding protein, putative	3	5	Both
ZP_02140643		probable binding protein component of ABC transporter	3	4	L
ZP_02139688		putative iron ABC transporter, periplasmic binding protein	3	6	Both
ZP_02141570		putative sugar ABC transporter, periplasmic sugar-binding protein	3	12	Both
ZP_02141905		putative sugar ABC transporter, periplasmic sugar-binding protein	2	3	D
ZP_02142183		solute-binding protein, putative	9	30	Both
ZP_02140549		spermidine/putrescine-binding periplasmic protein, putative	4	10	Both
ZP_02141152		sugar ABC transporter, periplasmic binding protein, putative	5	12	Both
ZP_02141492		sugar ABC transporter, periplasmic sugar-binding protein	5	11	Both
ZP_02140246		sugar ABC transporter, periplasmic sugar-binding protein, putative	5	21	Both
ZP_02142319		sugar ABC transporter, substrate-binding, putative	2	2	D
ZP_02140347		TRAP dicarboxylate ABC transporter, substrate-binding protein	6	13	Both
ZP_02142421		TRAP transporter solute receptor, TAXI family protein	3	9	Both
ZP_02138916		tungstate ABC transporter, tungstate-binding protein	1	3	D
ZP_02138986		branched-chain amino acid ABC transporter, periplasmic binding protein, putative	2	4	L

Accession No.	Gene	Protein	No. of Unique Peptides	Spectral Count	Expression[a]
ZP_02139318		branched-chain amino acid ABC transporter, periplasmic substrate-binding protein	2	11	Both
ZP_02143182		TRAP-type C4-dicarboxylate transport system protein, putative	3	3	D
ZP_02141634		TRAP-type transporter, putative	1	5	L
ZP_02140273		TRAP dicarboxylate transporter, DctP subunit, putative	5	15	Both
ZP_02141071		sugar ABC transporter protein, putative	8	25	Both
ZP_02141215		ABC transporter related protein	3	5	D
ZP_02141751		outer membrane porin, putative	21	203	Both
**Others**					
ZP_02139399	*secD*	protein-export membrane protein SecD, putative	2	3	D
ZP_02140400	*secB*	protein-export protein SecB, putative	3	4	Both
ZP_02138769	*tig*	trigger factor	3	8	Both
ZP_02140455	*secA*	preprotein translocase ATPase subunit	2	3	D
ZP_02140633	*dapD*	2,3,4,5-tetrahydropyridine-2-carboxylate N-succinyltransferase	1	3	L
ZP_02141057	*apbA*	2-dehydropantoate 2-reductase, putative	2	3	D
ZP_02140461	*cysQ*	3'(2'),5'-bisphosphate nucleotidase	2	2	D
ZP_02141896	*ats*	arylsulfatase	8	29	Both
ZP_02142699		bifunctional phosphopantothenoylcysteine decarboxylase/ phosphopantothenate synthase	1	3	D
ZP_02141463		dehydrogenase, putative	3	7	Both
ZP_02140970		formate--tetrahydrofolate ligase	7	17	Both
ZP_02140746		HflC protein, putative	7	27	Both
ZP_02140745		HflK protein, putative	3	8	Both
ZP_02140257		immunogenic protein, putative	7	14	Both
ZP_02143012		lipoprotein, putative	9	19	Both
ZP_02142241		L-sorbosone dehydrogenase, putative	3	6	Both
ZP_02142682		metallo-beta-lactamase family protein, putative	2	3	L
ZP_02141067		myo-inositol-1-monophosphotase	1	3	D
ZP_02139542		NAD(P) transhydrogenase subunit alpha	4	13	Both
ZP_02140465		sigma-54 modulation protein, putative	2	4	D
ZP_02142792		sulfatase, putative	5	17	Both
ZP_02141567		NifU-like domain protein	3	4	D
ZP_02139004		outer membrane protein, putative	2	3	L
ZP_02139355		outer membrane protein, putative	13	26	Both
ZP_02138809		peptidyl-prolyl cis-trans isomerase, cyclophilin-type, putative	2	4	D
ZP_02139426		peptidyl-prolyl cis-trans isomerse D, putative	2	4	D
ZP_02140454		PPIC-type PPIASE domain protein	2	4	L
ZP_02140138		quinone oxidoreductase	2	3	D
ZP_02141983		racemase, putative	3	7	Both
**Hyptothetical proteins**					
ZP_02140172		unkown protein	1	3	L
ZP_02140370		unkown protein	5	10	Both
ZP_02143493		unkown protein	4	14	Both
ZP_02142986		unkown protein	8	19	Both
ZP_02140398		unkown protein	5	12	Both
ZP_02143444		unkown protein	7	18	Both
ZP_02140608		unkown protein	10	25	Both
ZP_02140971		unkown protein	1	3	L
ZP_02141129		unkown protein	2	3	D
ZP_02141554		unkown protein	1	3	L
ZP_02141566		unkown protein	2	3	D
ZP_02141893		unkown protein	3	3	D
ZP_02142485		unkown protein	5	23	Both
ZP_02142561		unkown protein	4	6	D
ZP_02142651		unkown protein	2	3	L
ZP_02142704		unkown protein	1	3	L
ZP_02142653		unkown protein	4	12	Both
ZP_02142882		unkown protein	2	2	L
ZP_02143014		unkown protein	4	21	Both
ZP_02142987		unkown protein	2	2	L
ZP_02143024		unkown protein	3	4	D
ZP_02140098		unkown protein	5	14	Both
ZP_02140097		unkown protein	13	54	Both
ZP_02139612		unkown protein	7	25	Both
ZP_02138812		unkown protein	3	8	Both
ZP_02138766		unkown protein	2	3	D

[a] D means that the protein is solely expressed in dark condition; L means that the protein is solely expressed in light regimen condition; Both means that the protein is expressed under both conditions.

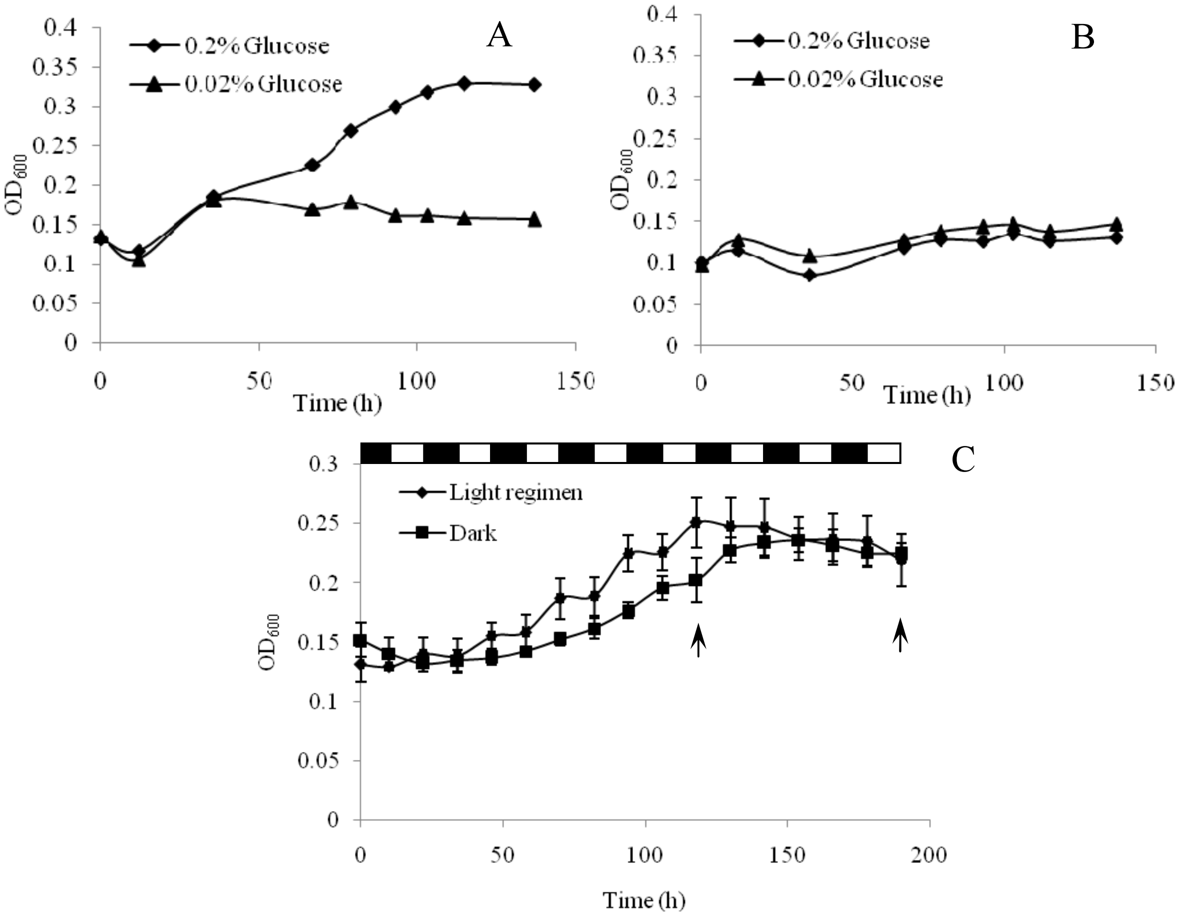

Fig. S1. Growth curves determined by measuring the optical density at 600 nm. **(**A, B) Growth curves of *R. litoralis* OCh149 (A) and *Erythrobacter litoralis* DSM8509 (B) in MMM containing 0.2% and 0.02% glucose. (C) Growth curves of *R. litoralis* OCh149 in MMM containing 0.2% glucose in both light regimen and dark conditions. The top zebra stripe indicates the dark:light (12 h:12 h) cycles of the light regimen. The arrows shows the sampling points for proteomic analysis.
